# Controlling residual hydrogen gas in mass spectra during pulsed laser atom probe tomography

**DOI:** 10.1186/s40679-017-0043-4

**Published:** 2017-02-22

**Authors:** R. Prakash Kolli

**Affiliations:** Department of Materials Science and Engineering, University of Maryland, 2144 Chemical and Nuclear Engineering Bldg., #090, College Park, MD 20742-2115 USA

**Keywords:** Atom probe tomography (APT), Copper, Hydrogen, Laser pulsing

## Abstract

Residual hydrogen (H_2_) gas in the analysis chamber of an atom probe instrument limits the ability to measure H concentration in metals and alloys. Measuring H concentration would permit quantification of important physical phenomena, such as hydrogen embrittlement, corrosion, hydrogen trapping, and grain boundary segregation. Increased insight into the behavior of residual H_2_ gas on the specimen tip surface in atom probe instruments could help reduce these limitations. The influence of user-selected experimental parameters on the field adsorption and desorption of residual H_2_ gas on nominally pure copper (Cu) was studied during ultraviolet pulsed laser atom probe tomography. The results indicate that the total residual hydrogen concentration, *H*_TOT_, in the mass spectra exhibits a generally decreasing trend with increasing laser pulse energy and increasing laser pulse frequency. Second-order interaction effects are also important. The pulse energy has the greatest influence on the quantity *H*_TOT_, which is consistently less than 0.1 at.% at a value of 80 pJ.

## Background

Residual hydrogen (H_2_) gas that is present in the analysis chamber of an atom probe instrument is typically detected in the mass spectrum of a specimen tip during analysis [1, 2]. The gas directly adsorbs to the specimen tip surface between each voltage or laser pulse and can then be desorbed during a subsequent pulse. The gas molecules impact the specimen tip and rebound thereby losing a small amount of kinetic energy during each bounce and then bind to a protruding surface atom [3]. The presence of field adsorbed residual H_2_ gas can limit the ability to quantify H concentration in metals and alloys during solid-state phenomena, such as hydrogen embrittlement, corrosion, hydrogen trapping, and grain boundary segregation since it is difficult to distinguish between the two sources of H. It also promotes the formation of metal hydride complex ions, M_*x*_H_*y*_, further complicating quantitative analysis of elemental concentrations in the mass spectra [1, 2]. Several recent studies have employed H charging where deuterium is introduced into the atom probe tomography (APT) specimen tip as a substitute for the H to be measured in an iron/vanadium (Fe/V) multilayer film [4], steels [5–7], silver (Ag) [7], zirconium (Zr) alloys [8], and silicon (Si) [9]. The quantity of residual H_2_ gas must, however, still be accounted for due to overlap in the mass spectra in order to estimate the H concentration. Hence, improved insight and understanding into the influence of experimental parameters on the behavior of residual H_2_ gas on the specimen tip surface in state-of-the-art atom probe instruments could help reduce these limitations. Such an understanding may permit minimization of the quantity of field adsorbed residual H_2_ gas and thereby allow more accurate quantification of the H concentration in metals and alloys.

Several authors have investigated the relationship between H_2_ gas adsorption and desorption and electric field during pulsed voltage one-dimensional (1D) atom probe field ion microscope (APFIM) analysis of materials including a nickel-rich (Ni-rich) alloy [10], tungsten (W) [11, 12], aluminum (Al) [13], and copper (Cu) [13]. Iron (Fe), Ni, Cu, palladium (Pd), molybdenum (Mo), W, and platinum (Pt) were also investigated as part of two broader studies [14, 15]. Some of the aforementioned studies illustrated that the H signal decreases with increasing field strength [11], or that a critical DC field strength exists depending on the metal or alloy, above which the H signal decreases with increasing field strength [10, 12].

Some studies on the effect of experimental parameters on H_2_ gas field adsorption and desorption using laser-pulsed mode in an atom probe instrument have been performed [16–18]. More specifically, field adsorption and desorption of H_2_ gas on molybdenum (Mo) during 1D-pulsed laser atom probe (PLAP) experiments equipped with a nitrogen laser (337 nm wavelength) was previously investigated [16]. This study evaluated the H signal intensity at substrate (base) temperatures between 100 and 150 K and at slow pulse rates between 1 and 60 Hz. An investigation on the field adsorption of H_2_ gas on W during 1D PLAP experiments was also performed [17] and evaluated the H signal intensity at temperatures between ~80 and 250 K. The pulse rates accessible by the instruments used in these studies are significantly slower than that attainable in today’s state-of-the-art instruments, and the base temperatures studied were greater than used today. More recently, the influence of laser pulse energy on field adsorption of residual H_2_ gas was evaluated on a nickel–chromium (Ni–Cr) alloy when using a Local-Electrode Atom-Probe (LEAP^®^) instrument equipped with a green laser (532 nm wavelength) [18].

The high mass resolving power, *m*/Δ*m*, and high elemental sensitivity in state-of-the-art atom probe instruments [19, 20] make performing APT measurements of H concentration in metals and alloys feasible. However, no studies have been performed to study the influence of user-selected experimental parameters in modern atom probe instruments equipped with a UV (355 nm wavelength) laser on the quantity of residual H_2_ gas detected in mass spectra. Furthermore, limited earlier studies using laser-pulsed mode were reported in the extant literature, and few have evaluated low temperatures <100 K, or fast pulse rates ≥100 kHz, or the combined effect of user-selected experimental parameters. Typically studies were performed to evaluate a single parameter. Additionally, Cu is an important electrical and structural material that experiences the aforementioned solid-state phenomena and studies on controlling residual H_2_ gas field adsorption and desorption on Cu have not been reported in the literature. Improved insight and understanding into the influence of experimental parameters in state-of-the-art atom probe instruments on the total residual hydrogen concentration, *H*_TOT_, that is detected may permit minimization of the gas in mass spectra. This study evaluates the synergistic laser pulse energy, laser pulse frequency, and specimen tip base temperature effects on the quantity *H*_TOT_ that is detected during UV-pulsed laser APT of nominally pure Cu. The results are discussed in context of a constant evaporation rate theoretical model in order to evaluate the relative influence of the three aforesaid parameters. A statistical analysis was also performed to ascertain the significance of each parameter and their interactions on the quantity *H*_TOT_. Other H sources that can influence the quantity *H*_TOT_ are discussed including the possibility of impingement and dissociation of H_2_O on the specimen tip and surface diffusion of residual H_2_ gas molecules. The short-range binding energy of a H_2_ molecule on a Cu metal surface atom is estimated based on the model presented in Refs. [21, 22], and the mechanisms to form the H^+^ and ${\text{H}}_{3}^{ + }$ ions detected in the mass spectra are discussed in context of the binding energy. We discuss how the residual H_2_ gas in the mass spectra can be minimized and the broader applicability of the results to other materials.

## Experimental methods

A 0.5 mm diameter 99.999 wt.% polycrystalline Cu wire taken from Standard Reference Material (SRM) 482 of the National Institute of Standards and Technology (NIST) was used as the source material. Specimens with a needle-shaped geometry necessary for APT analysis were fabricated using a FEI Nova 600 dual-beam scanning electron microscope/focused ion beam (SEM/FIB) instrument following standard lift-out and annular milling procedures [23–25]. A platinum (Pt) protective layer was deposited over a region of interest (ROI) using established procedures. Annular milling was performed employing a 30 kV gallium ion (Ga^+^) beam and sequentially decreasing probe current following standard procedures after transfer of the ROI to silicon (Si) microtip posts with an Omniprobe Autoprobe 200 Micromanipulator. A low kV ion beam of 5 kV was allowed to raster over the specimen tip to remove material that had been damaged by the 30 kV Ga^+^ ion beam annular milling operation [26].

Pulsed laser APT was performed employing a CAMECA LEAP^®^ 4000X instrument in the Si configuration with a 355 nm UV-pulsed laser under ultra-high vacuum (UHV) conditions of 1.2 × 10^−8^ Pa (9 × 10^−11^ Torr). A full factorial 3 × 3 × 3 experimental matrix was used where the laser pulse energy was set at 40, 60, or 80 pJ; the laser pulse frequency at 100, 250, or 500 kHz; and the specimen tip base temperature at 20, 50, or 80 K leading to 27 individual experiments on different specimen tips. The nominal detection rate was maintained at a constant 0.01 ions per pulse or 1%. Datasets of greater than two million ions were collected for each experiment. Mass spectra analysis was performed using the CAMECA Integrated Visualization and Analysis Software (IVAS^®^), version 3.6.6. Statistical analysis of the data by analysis of variance (ANOVA) using the *F* test with a significance of *α* = 0.05 was performed employing the JMP^®^ software, version 12.

## Results

### Mass spectrum

**Fig. 1:**
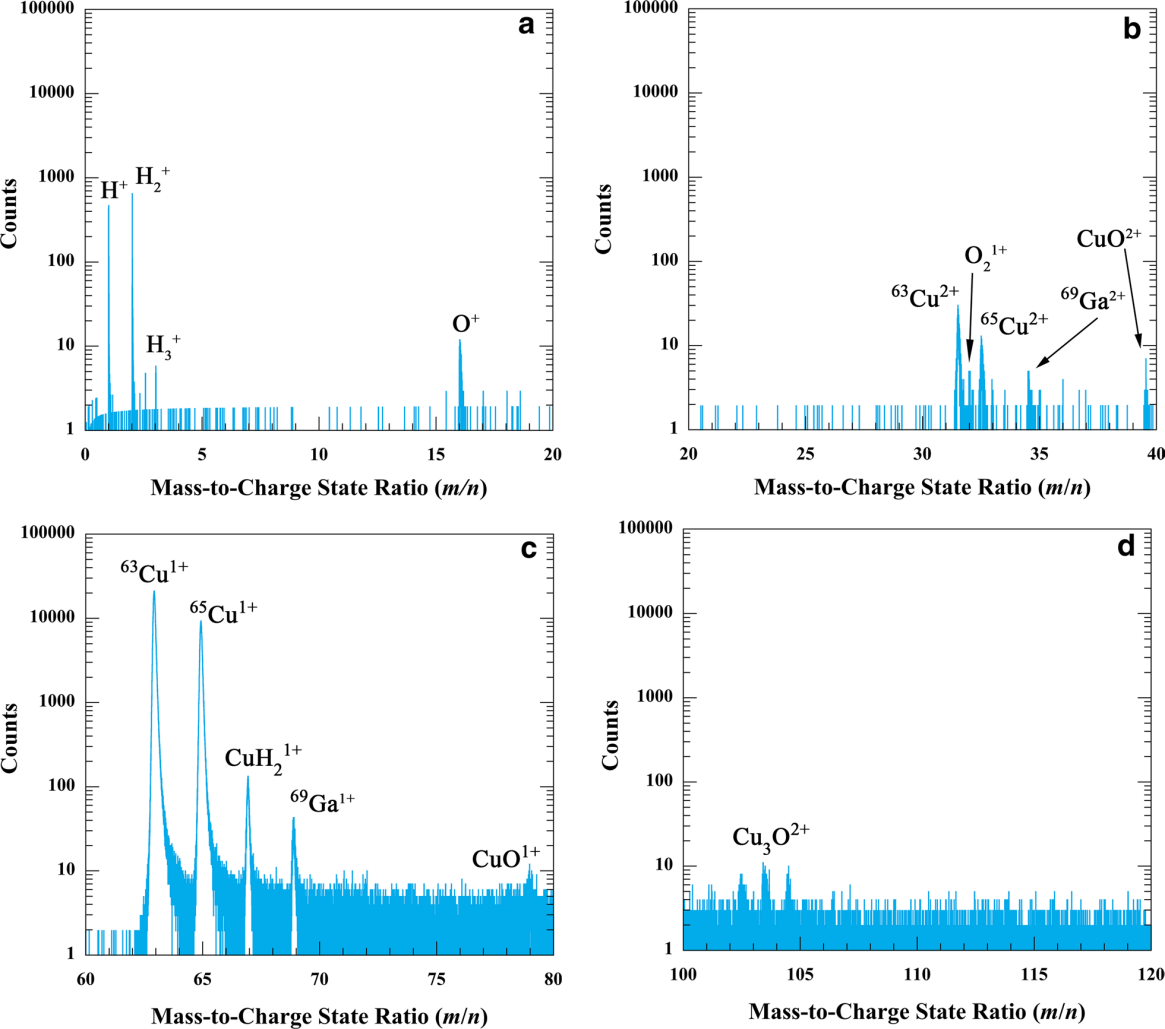
Sections of an example mass spectrum illustrate the presence of **a** H^+^, ${\text{H}}_{2}^{ + }$, ${\text{H}}_{3}^{ + }$, and O^+^ peaks; **b** Cu^2+^, ${\text{O}}_{2}^{1 + }$, Ga^2+^, and CuO^2+^ peaks; **c** Cu^+^, ${\text{CuH}}_{ 2}^{ 1+ }$, Ga^+^, and CuO^1+^ peaks; and **d** Cu_3_O^2+^ peaks. The laser pulse energy is 40 pJ, the laser pulse frequency is 100 kHz, and the specimen tip base temperature is 20 K

Sections of a background-noise-corrected example mass spectrum are illustrated in Fig. 1a–d. Hydrogen is present as atomic hydrogen, H^+^, at a mass-to-charge state (*m*/*n*) ratio of 1 Da, and also as molecular hydrogen, ${\text{H}}_{2}^{ + }$ and ${\text{H}}_{3}^{ + }$, at *m*/*n* ratios of 2 and 3 Da. The peak at a *m*/*n* ratio of 1 Da may also contain ${\text{H}}_{2}^{2 + }$ ions. The ${\text{H}}_{3}^{ + }$ peak is small relative to the H^+^ and ${\text{H}}_{2}^{ + }$ peaks in this example mass spectrum but can have a greater number of counts depending on the user-selected experimental parameters. Hydrogen is also present as a complex ion at a *m*/*n* ratio of 67 Da, which we have ascribed to ${\text{CuH}}_{ 2}^{ 1+ }$. This complex ion should also be present at a *m*/*n* ratio of 65 Da but it is convoluted with ^65^Cu^1+^. Oxygen (O) is present at a *m*/*n* ratio of 16 Da and as molecular oxygen, ${\text{O}}_{2}^{1 + }$, at a *m*/*n* ratio of 32 Da. Oxygen is also present as part of complex ions at *m*/*n* ratios of 39.5 and 79 Da, which we have ascribed to CuO^2+^ and CuO^1+^, respectively, and at *m*/*n* ratios of 102.5, 103.5, and 104.5 Da, which we have ascribed to Cu_3_O^2+^. The complex ions were identified through ion correlation histograms, which are two-dimensional (2D) histograms of the ion pairs in multi-hit ion detection events [27, 28], and illustrated for Cu in Refs. [29, 30]. Additionally, the expected peaks for Cu at *m*/*n* ratios of 63 and 65 Da for the 1+ charged state and at *m*/*n* ratios of 31.5 and 32.5 Da for the 2+ charged states are present. Gallium is present in small quantities at *m*/*n* ratios of 69 and 34.5 Da for the 1+ and 2+ charged states, respectively.

### Hydrogen

**Fig. 2:**
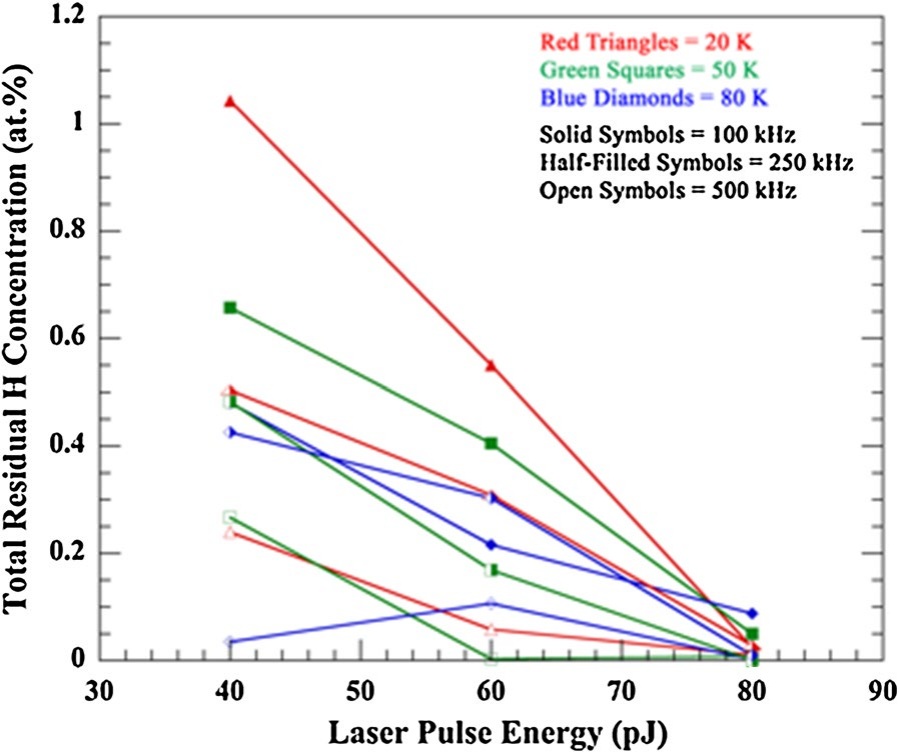
The total residual H concentration, *H*_TOT_, (at.%) plotted as a function of laser pulse energy between 40 and 80 pJ. The specimen tip base temperatures are 20 K (*red triangles*), 50 K (*green squares*), or 80 K (*blue diamonds*), and the laser pulse frequencies are 100 kHz (*solid symbols*), 250 kHz (*half-filled symbols*), and 500 kHz (*open symbols*). The ±2*σ* *error bars*, which are based on counting statistics, are not visible since they are smaller than the size of the data point markers

The quantity *H*_TOT_ is the sum of the atomic, molecular, and complex ion hydrogen and thus includes H^+^, ${\text{H}}_{2}^{ + }$, ${\text{H}}_{3}^{ + }$, and ${\text{CuH}}_{ 2}^{ 1+ }$. All the ions at a *m*/*n* ratio of 1 Da are assigned to H^+^ since the detected quantity of ${\text{H}}_{2}^{2 + }$ is expected to be minimal, as discussed below. Since hydrogen in the ${\text{CuH}}_{ 2}^{ 1+ }$ complex ion at a *m*/*n* ratio of 65 Da is convoluted with ^65^Cu^1+^, the quantity of H at this peak contributing to the quantity *H*_TOT_ is determined based upon the isotopic abundance ratio of Cu, i.e., ${\text{CuH}}_{ 2}^{ 1+ }$ is detected in the same abundance ratio as Cu. Measureable quantities of H are still present in the mass spectra despite performing the experiments at UHV typical of state-of-the-art atom probe analysis. The quantity *H*_TOT_ in the mass spectra is illustrated as a function of laser pulse energy at specimen tip base temperatures of 20 K (red triangles), 50 K (green squares), and 80 K (blue diamonds); and laser pulse frequencies of 100 kHz (solid symbols), 250 kHz (half-filled symbols), and 500 kHz (open symbols), Fig. 2. The quantity *H*_TOT_ demonstrates a decreasing trend between 40 and 80 pJ pulse energies at these base temperatures and pulse frequencies of 100 and 250 kHz. The relationship is more complex at a pulse frequency of 500 kHz. At a base temperature of 20 K, a decreasing trend is observed in the quantity *H*_TOT_ between 40 and 80 pJ. At a base temperature of 50 K, a decreasing trend in the quantity *H*_TOT_ is observed between 40 and 60 pJ, and it is then approximately constant to 80 pJ. More variability is observed when the base temperature is 80 K. Further, the quantity *H*_TOT_ decreases with increasing pulse frequency at pulse energies of 40 and 60 pJ and at a base temperature of 20 and 50 K but some variability is again observed at a temperature of 80 K. Less difference is exhibited in the quantity *H*_TOT_ between frequencies at a pulse energy of 80 pJ.

**Fig. 3:**
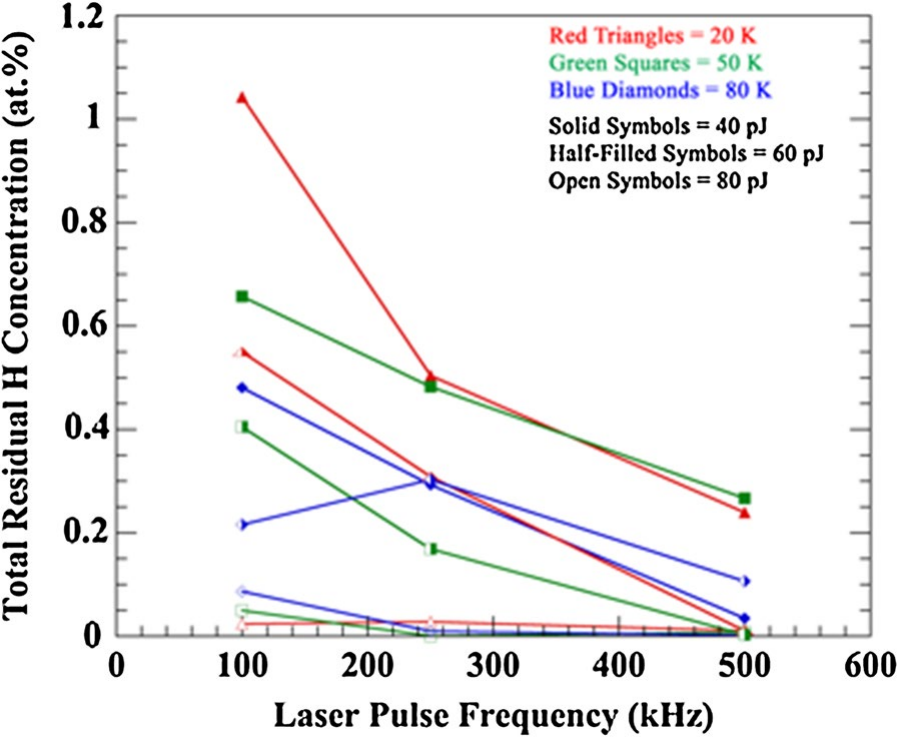
The total residual H concentration, *H*_TOT_, (at.%) plotted as a function of laser pulse frequency between 100 and 500 kHz. The specimen tip base temperatures are 20 K (*red triangles*), 50 K (*green squares*), or 80 K (*blue diamonds*), and the laser pulse energies are 40 pJ (*solid symbols*), 60 pJ (*half-filled symbols*), and 80 pJ (*open symbols*). The ±2*σ* *error bars*, which are based on counting statistics, are not visible since they are smaller than the size of the data point markers

The quantity *H*_TOT_ is also illustrated as a function of laser pulse frequency at specimen tip base temperatures of 20 K (red triangles), 50 K (green squares), and 80 K (blue diamonds) and at laser pulse energies of 40 pJ (solid symbols), 60 pJ (half-filled symbols), and 80 pJ (open symbols), Fig. 3. The quantity *H*_TOT_ demonstrates a decreasing trend with increasing pulse frequency at a pulse energy of 40 and 60 pJ and at these base temperatures, although some variability is observed at 80 K. The pulse frequency has a much smaller effect on the quantity *H*_TOT_ at a pulse energy of 80 pJ, which is approximately constant between 250 and 500 kHz at all three base temperatures. A slightly decreasing trend is, however, observed with increasing frequency between 100 and 250 kHz at 50 and 80 K, whereas it is approximately constant at 20 K. Additionally, the specimen tip base temperature has less effect on the quantity *H*_TOT_ at an 80 pJ pulse energy when compared to 40 and 60 pJ. Increasing the pulse energy at a pulse frequency of 100 or 250 kHz and a given base temperature reduces the quantity *H*_TOT_. The relationship is more complex at a frequency of 500 kHz.

**Fig. 4:**
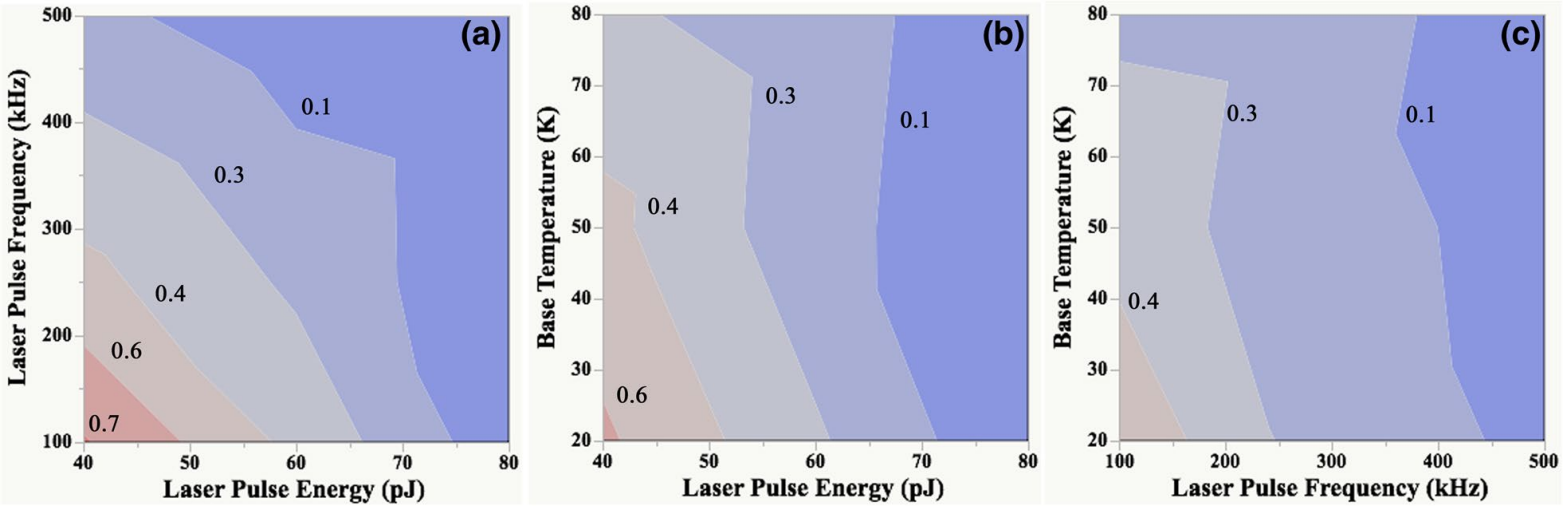
Contour plots illustrating the interaction of the experimental parameters **a** laser pulse frequency and laser pulse energy, **b** base temperture and laser pulse energy, and **c** base temperature and laser pulse frequency at a constant value of total residual H concentration, *H*_TOT_, (at.%). Lower values of the quantity *H*_TOT_ are represented by cool coloration (*purple and blue*) and higher values are represented by warm coloration (*pink and red*)

In order to determine the significance of each experimental parameter, ANOVA was performed on the data with the quantity *H*_TOT_ as the response variable. The observed nonlinearities and variability at certain experimental parameter combinations in Figs. 2 and 3 suggest that complex interactions between the parameters may also influence the quantity *H*_TOT_, and these were also evaluated. The main experimental parameters of laser pulse energy (*F* = 137.1, *P* < 0.001), laser pulse frequency (*F* = 68.9, *P* < 0.001), and base temperature (*F* = 10.9, *P* < 0.004) were significant. Additionally, ANOVA illustrated as significant the second-order interactions of pulse energy and pulse frequency (*F* = 30.2, *P* < 0.001), pulse energy and base temperature (*F* = 10.4, *P* < 0.001), and pulse frequency and base temperature (*F* = 4.7, *P* < 0.042). Contour plots of the experimental parameters illustrate their complex interaction at constant values of *H*_TOT_, Fig. 4a–c. The plots also illustrate the dependence of the quantity *H*_TOT_ on the experimental parameters.

## Discussion

### Influence of the experimental parameters on total residual H concentration

The results indicate that laser pulse energy has greater influence on the quantity *H*_TOT_ when compared to laser pulse frequency and specimen tip base temperature. Increasing the laser pulse energy from 40 to 80 pJ significantly lowers the quantity *H*_TOT_. At a 80 pJ pulse energy, the quantity *H*_TOT_ is consistently less than 0.1 at.% regardless of the choice of pulse frequency or base temperature within the studied range of experimental parameters. This value is lower than the ~0.2 at.% attained for the total H concentration when employing a green laser at ~600 pJ in Ref. [18]. Increasing the laser pulse frequency from 100 to 500 kHz also lowers the quantity *H*_TOT_. The approximately linear relationship between the quantity *H*_TOT_ and increasing laser pulse frequency at a 40 pJ pulse energy and at a 50 K base temperature and also at 60 pJ and 20 or 50 K are consistent with the observations between 1 and 60 Hz in Ref. [16], and 50 and 200 kHz in Ref. [18] thereby indicating that the reported linear proportionality extends to higher pulse frequencies. However, a similar linear relationship is not observed when the pulse energy is 80 pJ, which may be due to its greater influence and second-order interaction effects. Further, the predominant effect of pulse frequency during field adsorption is to affect the time available for adsorption of residual H_2_ gas molecules between each pulse, i.e., a faster pulse frequency will reduce the time available. Hence, a faster pulse frequency will lead to a lower amount of surface coverage of an adsorbing gas than at a slower pulse frequency thereby leading to a lower value for the quantity *H*_TOT_. The base temperature has the least influence on the quantity *H*_TOT_, especially when the pulse energy is 80 pJ, and the pulse frequency is 500 kHz.

The approximately constant relationship or only small variation of the quantity *H*_TOT_ as a function of pulse frequency at a 80 pJ pulse energy, and a given specimen tip base temperature suggests a change in physical behavior that is possibly related to the greater influence of pulse energy. The measured dependence of the quantity *H*_TOT_ on the experimental parameters suggests a relationship between these parameters and the specimen tip apex temperature at a constant evaporation rate. The evaporation rate at time *t*, $\varPhi \left( t \right)$, due to the electric field strength derived from the applied DC voltage, *F*_0_, specimen tip base temperature, *T*_0_, and temperature rise of the specimen tip apex due to laser pulsing, *T*_rise_, can be expressed as [31]

1 $$\varPhi \left( t \right) \approx \upsilon N\tau_{\text{t}} \exp \left( {\frac{{ - Q_{n} \left( {F_{0} } \right)}}{{k_{\text{B}} \left( {T_{0} + T_{\text{rise}} } \right)}}} \right),$$

where *ν* is the vibrational energy of the *N* kink site atoms that can be field evaporated, *τ*_t_ is the temporal width of the temperature profile, *Q*_*n*_ is the activation energy that is a function of *F*_0_, and *k*_B_ is Boltzmann’s constant. At a constant evaporation rate, the field strength is greater in value at a lower specimen tip apex temperature, *T*_0_ + *T*_rise_, that are characteristic of low-pulse energies. Conversely, the field strength is lower in value at a higher tip apex temperature that are characteristic of high-pulse energies since the quantity *T*_rise_ increases with increasing laser pulse energy [32–35]. Thus, since the quantity *H*_TOT_ is related to the quantity *F*_0_ [16, 18], it is also related to the quantity *T*_0_ + *T*_rise_ through Eq. (1). The quantity *T*_rise_ has been estimated as between 160 and 300 K during normal APT operating conditions [34, 36], which is greater than the quantity *T*_0_ and hence suggests that the laser pulse energy has a greater influence on the quantity *H*_TOT_.

The second-order interaction between laser pulse energy and laser pulse frequency is also important in reducing the quantity *H*_TOT_, which is possibly due to both parameters influence on the specimen tip apex temperature combined with the latter’s effect on residual H_2_ gas surface coverage. We discuss above the pulse energy effect on tip apex temperature and pulse frequency may also affect it since it directly correlates to cooling time between pulses [29, 30]. The second-order interactions between base temperature and laser pulse energy and also laser pulse frequency are less important and have less effect on reducing the quantity *H*_TOT_.

The combination of a 80 pJ laser pulse energy, 500 kHz laser pulse frequency, and a base temperature between 20 and 80 K leads to a value for the quantity *H*_TOT_ <0.1 at.%. The combination of a 80 pJ laser pulse energy, 250 kHz laser pulse frequency, and 20 K specimen tip base temperature leads to a value for the quantity *H*_TOT_ <0.1 at.%, and also improved mass resolving power and smaller tails after major single-charged state peaks in Cu based on prior results in Refs. [29, 30].

### Residual H_2_ gas supply mechanism

Three sources of residual H_2_ gas that adsorb to the APT specimen tip may occur: (1) direct adsorption of H_2_ gas, (2) impingement and dissociation of H_2_O molecules, and possibly (3) surface diffusion of H_2_ gas along the shank of the tip due to a thermal or field gradient as suggested in Refs. [10, 18]. Direct adsorption of residual H_2_ gas from the analysis chamber is addressed above. In addition, the experimental observations suggest that small quantities of H_2_O impingement and dissociation may be exhibited in some specimen tips. Although a H_2_O^+^ peak is not observed at a *m*/*n* ratio of 18 in Fig. 1a, this peak is present in the mass spectrum of some specimen tips. Furthermore, a OH^+^ peak is observed in the mass spectrum at a *m*/*n* ratio of 17 indicating that dissociation of H_2_O is occurring. In addition to the complex ions consisting of Cu, H, O, and O_2_ combinations as described above, complex ion peaks at *m*/*n* ratios of 80 and 82 that correspond to CuOH^+^ are also observed in the mass spectrum when H_2_O impingement and dissociation occurs. The contribution of this source to the quantity *H*_TOT_ is, however, small when compared to that of direct adsorption of H_2_ gas.

The experimental observations preclude the possibility of significant residual H_2_ gas surface diffusion along the shank of the tip from its base to its apex due to a thermal gradient. To a first approximation, surface diffusion obeys Arrhenius behavior where the diffusivity (or the diffusion coefficient) increases with increasing temperature as

2 $$D = D_{0} { \exp }\left( {\frac{{ - Q_{\text{S}} }}{RT}} \right),$$

where *D*_0_ is the pre-exponential constant; *Q*_S_ is the activation energy for surface diffusion; *R* is the gas constant; and *T* is the absolute temperature. If surface diffusion due to thermal gradient was a significant mechanism to supply H_2_ gas to the tip apex, the quantity *H*_TOT_ should increase with increasing pulse energy at a given specimen tip base temperature and pulse frequency, which is not observed in the results. Alternatively, the increase in temperature at the specimen tip apex due to increased laser pulse energy concomitantly reduces the field at this location and thus the field gradient between the tip apex and its base. This in turn may cause a reduction in the supply of residual H_2_ gas by surface diffusion to the tip apex that corresponds to a reduction in the quantity *H*_TOT_. The field strength at the specimen tip base and shank is, however, less than at the tip apex thereby suggesting that limited residual H_2_ gas adsorbs at these locations. Residual H_2_ gas may also be adsorbed at the specimen tip base and shank due to trapping at cryogenic temperatures, i.e., a “cold finger” mechanism. But significant quantities of H_2_ are not trapped until temperatures are less than ~10 K, which is lower than the specimen tip base temperatures in this study. Thus, surface diffusion along the shank of the tip from its base to its apex due to field gradient most likely has limited contribution to the residual H_2_ gas detected in a mass spectrum and the quantity *H*_TOT_ when compared to that of direct adsorption of H_2_ gas.

### Short-range binding energy

The short-range binding energy is estimated employing the model developed by Tsong and Müller [21, 22]. These authors suggested that field adsorption occurs due to short-range field-induced dipole–dipole interactions between the gas molecules and the apex of protruding metal surface atoms, where short-range is defined as an atomic radius or less [21, 22]. This mechanism means that the protruding metal surface atoms, whether kink site or step site atoms, and the adsorbing species are polarizable due to the applied DC electric field. In this model, the binding energy, H_A_, is

3 $$\frac{1}{2}\alpha_{\text{A}} \left( {f_{\text{A}} - 1} \right)F_{0}^{2} ,$$

where *α*_A_ is the polarizability of the gas atom or molecule; *f*_A_ is the enhancement factor due to field-induced dipole–dipole interactions; and *F*_0_ is the applied electric field strength in V/Å. The quantity *f*_A_ is given by

4 $$f_{\text{A}} = \frac{{\left( {1 + {{2\alpha_{\text{M}} } \mathord{\left/ {\vphantom {{2\alpha_{\text{M}} } {d^{3} }}} \right. \kern-0pt} {d^{3} }}} \right)^{2} }}{{\left( {1 - {{4\alpha_{\text{M}} \alpha_{\text{A}} } \mathord{\left/ {\vphantom {{4\alpha_{\text{M}} \alpha_{\text{A}} } {d^{6} }}} \right. \kern-0pt} {d^{6} }}} \right)^{2} }},$$

where *α*_M_ is the polarizability of the metal surface atom, and *d* is the equilibrium distance of the interacting surface atom and the adsorbing species. The polarizability is related to the Gaussian or static electric dipole polarizability by the factor 4*πɛ*_0_, where *ɛ*_0_ is the vacuum permittivity in free space. This model is used here since it reportedly has good correlation to experimentally measured values of short-range binding energies [37, 38], despite its differences in underlying theory with the image dipole model developed by Forbes [37–39], and the combination of short-range field-induced polarization bonding and field-induced covalent bonding model developed by Krezuer [38].

**Fig. 5:**
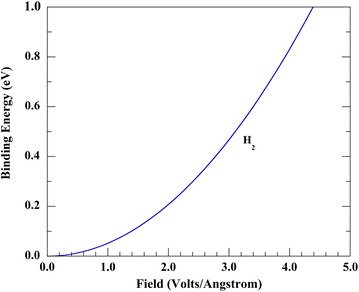
The estimated short-range binding energy for field adsorbed residual H_2_ gas (*blue line*) on a copper (Cu) metal surface atom as a function of the field strength

The short-range binding energy of a H_2_ gas molecule above a Cu metal surface atom is estimated using Eqs. (3) and (4). These equations assume that mutual polarization between adjacent dipoles in the gas and metal substrate and secondary interactions between the adsorbing species and surface atoms are negligible. These factors lead to only a small improvement of the short-range binding energy calculations in this model [37]. Additionally, the above equations assume that the kinetic energy gained by the ions as they move from a distant point to their binding point is negligible. This factor also leads to only a small enhancement of the short-range binding energy calculations in this model [37]. Thus, the first-order approximation that employs Eqs. (3) and (4) is a reasonable one to estimate the short-range binding energy of a H_2_ gas molecule on a Cu metal surface atom. The Gaussian polarizability of Cu, *α*_Cu_, is 5.0 Å^3^ [40], the atomic radius of Cu, *r*_Cu_, is 0.135 nm [41], and the Gaussian polarizability of H_2_, $\alpha_{{{\text{H}}_{2} }}$, is 0.79 Å^3^ [16]. The calculated short-range binding energy for a H_2_ molecule as a function of the field strength after converting the Gaussian polarizability to standard SI units is illustrated in Fig. 5, where *d* = *r*_Cu_ + *r*_H_ = 0.255 nm. An earlier estimate of the field strength that is required for field-induced adsorption of a H_2_ molecule on a Cu surface atom during pulsed voltage 1D APFIM was 2.05 V/Å [13]. This field strength value suggests that the short-range binding energy is approximately 0.21 eV using Fig. 5.

### ${\text{H}}^{ + }$ ion dissociation and ${\text{H}}_{3}^{ + }$ ions

The 0.21 eV value for the quantity H_A_ of a H_2_ molecule on a Cu surface atom is approximately an order of magnitude less than the reported binding energy of between 2.0 and 3.3 eV for a H atom on a Cu surface atom [42, 43]. The high values for the atomic H binding energy suggests that it would not be easily desorbed from the specimen tip during the thermal pulse. Since the quantity H_A_ of molecular H_2_ is significantly less than the binding energy of atomic H, it is likely that the H^+^ ions detected in the mass spectrum at a *m*/*n* ratio of 1 Da occurred due to desorption of ${\text{H}}_{2}^{ + }$ ions followed by ionization and dissociation of a subset of these ions in the high field surrounding the tip apex, i.e., ${\text{H}}_{2}^{ + } \to {\text{H}}_{2}^{2 + } \to {\text{H}}^{ + } + {\text{H}}^{ + }$.

The presence of ${\text{H}}_{3}^{ + }$ ions in the example mass spectrum of Fig. 1a requires further discussion. Although neutral H_3_ is unstable in free space, it may be stable in the presence of an electric field. The ${\text{H}}_{3}^{ + }$ ion reportedly has a stable configuration in the electric field and after desorption [44, 45]. It also does not easily field dissociate until a field strength of approximately 3.0 V/Å [44, 45], and thus, it can be detected in the mass spectra. Furthermore, the quantity of measured ${\text{H}}_{3}^{ + }$ ions depends on the metal surface atom’s crystallographic structure, field strength, laser pulse energy, and the specimen tip apex temperature [1, 46, 47]. For example, elements with a face-centered cubic (f.c.c.) crystal structure, such as Cu, exhibit lower quantities of ${\text{H}}_{3}^{ + }$ ions in a mass spectrum when compared to elements with hexagonal close-packed (h.c.p.) and body-centered cubic (b.c.c.) crystal structures. The experimental observations in this study are consistent with those in Refs. [1, 46, 47] that indicate the quantity of ${\text{H}}_{3}^{ + }$ ions measured in the mass spectra is dependent on the experimental parameters. For example, approximately 223 ${\text{H}}_{3}^{ + }$ ions are measured when the laser pulse energy is 40 pJ, and ca. 0 ${\text{H}}_{3}^{ + }$ ions when the pulse energy is 80 pJ, at a laser pulse frequency of 250 kHz and specimen tip base temperature of 20 K. The occurrence of the ${\text{H}}_{3}^{ + }$ ion in mass spectra during PLAP has been attributed to direct adsorption on highly protruded kink or step site atoms due to the aforementioned stability in the presence of an electric field followed by ionization and desorption [1, 46–48]. Alternatively, the formation of the ${\text{H}}_{3}^{ + }$ ion has been suggested to occur by migration of the more weakly bound H_2_ molecule that hops onto a more strongly bound H atom thereby forming a H_3_ species that is ionized and desorbed as a ${\text{H}}_{3}^{ + }$ ion [44, 49]. However, in view of the limited expected surface diffusion at the tip apex until its temperature attains ~800 K [33–35], the estimated short-range binding energy, and the aforesaid ionization, desorption, and dissociation model of the ${\text{H}}_{2}^{ + }$ ion suggest that the model described in Refs. [1, 46–48] may be correct.

### Minimizing residual H_2_ gas in mass spectra and applicability to other materials

In general, this study illustrates that choosing the appropriate values of user-selected experimental parameters of laser pulse energy, laser pulse frequency, and specimen tip base temperature can appreciably minimize the quantity of residual H_2_ gas in the mass spectrum. The results of this study indicate that by increasing the laser pulse energy at a constant laser pulse frequency and specimen tip base temperature, the quantity of residual H_2_ gas in the mass spectrum can be reduced to values less than 0.1 at.%. Moreover, the results of this study indicate that by increasing the laser pulse frequency at a constant laser pulse energy and base temperature, the quantity of residual H_2_ gas in the mass spectrum can be reduced. Alternatively, both pulse energy and pulse frequency can be increased at a constant base temperature to attain low values of the quantity *H*_TOT_. Virtually all metal and alloy APT mass spectra will exhibit H and the results of this study on Cu are likely broadly applicable. The few earlier studies that evaluated single parameters in laser-pulsed mode on other metals or alloys support this observation. One previous study on Mo indicated that the H signal intensity decreases with increasing pulse frequency albeit at significantly slower rates [16]. Furthermore, a second previous study on a Ni–Cr-rich alloy indicated that the quantity of adsorbed H decreases with increasing pulse energy. The applicability to materials other than metals and alloys, such as semi-conducting materials, has not been examined in detail and needs further study in order to gain greater understanding of the behavior. For example, the APT mass spectra of cadmium telluride (CdTe) in one recent study did not exhibit H [50, 51]. Furthermore, the study in Ref. [51] suggested the opposite trend for residual H_2_ gas in the mass spectra with laser pulse energy in gallium nitride (GaN). The differences seen in these materials when compared to metals and alloys may be due to material specific properties of these compound semiconductors.

## Conclusions

The presence of residual H_2_ gas in the mass spectra limits the ability to measure H concentration in metals and alloys in solid-state phenomena using APT since it is difficult to distinguish between the sources of H and thus needs to be minimized. The synergistic laser pulse energy, laser pulse frequency, and specimen tip base temperature influence on the field adsorption and desorption of residual H_2_ gas on nominally pure Cu was studied during UV-pulsed laser APT. This investigation resulted in the following findings:

1. Statistical analysis by ANOVA demonstrates that laser pulse energy, laser pulse frequency, and specimen tip base temperature have a significant effect on the total residual H concentration, *H*_TOT_. Second-order interactions of pulse energy and pulse frequency, pulse energy and base temperature, and pulse frequency and base temperature are also shown to be significant.
2. The quantity *H*_TOT_ exhibits a decreasing trend with increasing laser pulse energy between 40 and 80 pJ at laser pulse frequencies of 100, 250, and 500 kHz at a constant specimen tip base temperature of 20 or 50 K.
3. The quantity *H*_TOT_ exhibits a decreasing trend with increasing pulse frequency between 100 and 500 kHz at pulse energies of 40 and 60 pJ and at base temperatures of 20 or 50 K. The quantity *H*_TOT_ exhibits a generally decreasing or constant trend with increasing pulse frequency at a pulse energy of 80 pJ and at base temperatures of 20, 50, and 80 K.
4. Laser pulse energy has the greatest effect on the quantity *H*_TOT_, which is less than 0.1 at.% at a pulse energy of 80 pJ, and pulse frequencies of 100, 250, or 500 kHz, and base temperatures of 20, 50, or 80 K. The pulse frequency and base temperature have only a smaller effect on the quantity *H*_TOT_ at a 80 pJ pulse energy when compared to a 40 or 60 pJ pulse energy.
5. The results of this study indicate that the quantity of residual H_2_ gas can be minimized in mass spectra by increasing the laser pulse energy at a constant laser pulse frequency or specimen tip base temperature. The quantity of residual H_2_ gas can also be minimized in mass spectra by increasing the laser pulse frequency at a constant laser pulse energy and base temperature. Alternatively, both pulse energy and pulse frequency can be increased at a constant base temperature.

## Abbreviations

APT: atom probe tomography
UV: ultraviolet
1D: one-dimensional
APFIM: atom probe field ion microscope
PLAP: pulsed laser atom probe
LEAP: local-electrode atom probe
UHV: ultra-high vacuum
IVAS: integrated visualization and analysis software
ANOVA: analysis of variance
2D: two-dimensional
f.c.c.: face-centered cubic
b.c.c.: body-centered cubic
h.c.p.: hexagonal close-packed

## References

[1] Tsong TT, Kinkus TJ (1984). Energy distributions of pulsed-laser field-desorbed gaseous ions and field-evaporated metal ions: a direct time-of-flight measurement. Phys. Rev. B..

[2] Gault B, Moody MP, Cairney JM, Ringer SP (2012). Atom Probe Microscopy.

[3] Larson DJ, Prosa TJ, Ulfig RM, Geiser BP, Kelly TF (2013). Local Electrode Atom Probe Tomography—A User’s Guide.

[4] Gemma R, Al-Kassab T, Kirchheim R, Pundt A (2012). Visualization of deuterium dead layer by atom probe tomography. Scripta Mater..

[5] Takahashi J, Kawakami K, Kobayashi Y, Tarui T (2010). The first direct observation of hydrogen trapping sites in TiC precipitation-hardening steel through atom probe tomography. Scripta Mater..

[6] Takahashi J, Kawakami K, Tarui T (2012). Direct observation of hydrogen-trapping sites in vanadium carbide precipitation steel by atom probe tomography. Scripta Mater..

[7] Haley D, Merzlikin SV, Choi P, Raabe D (2014). Atom probe tomography observation of hydrogen in high-Mn steel and silver charged via an electrolytic route. Int. J. Hydrogen Energy.

[8] Sundell G, Thuvander M, Yatim AK, Nordin H, Andrén H-O (2015). Direct observation of hydrogen and deuterium in oxide grain boundaries in corroded zirconium alloys. Corros. Sci..

[9] Takamizawa H, Hoshi K, Shimizu Y, Yano F, Inoue K, Nagata S, Shikama T, Nagai Y (2013). Three-dimensional characterization of deuterium implanted in silicon using atom probe tomography. Appl. Phys. Express.

[10] Andrén H-O, Rolander U (1992). Field dependence of hydrogen adsorption. Surf. Sci..

[11] Macrander AT, Seidman DN (1984). Hydrogen adsorption on (110) tungsten at 30 k: an atom-probe field-ion microscope study. Surf. Sci..

[12] Hellsing M, Hellsing B (1986). Field adsorption and desorption of hydrogen on W(110)—an atom-probe study. Surf. Sci..

[13] Nishikawa O, Yoshimura T, Shibata M (1983). Atom-probe study of hydrogen physisorption on Al and Cu. Surf. Sci..

[14] Wada M, Uemori R, Nishikawa O (1983). Effect of hydrogen on the evaporation field of metals. Surf. Sci..

[15] Nishikawa O (1983). Reduced evaporation field by the field induced dipoles of physisorbed He, Ne and H_2_. Surf. Sci..

[16] Kellogg GL (1981). Pulsed laser stimulated field desorption of hydrogen from molybdenum. J. Chem. Phys..

[17] Drachsel W, Block JH (1991). Isotopic effects in field desorption of hydrogen from tungsten. Surf. Sci..

[18] Sundell G, Thuvander M, Andrén H-O (2013). Hydrogen analysis in APT: methods to control adsorption and dissociation of H_2_. Ultramicroscopy.

[19] Kelly TF, Larson DJ (2012). Atom probe tomography 2012. Annu. Rev. Mater. Res..

[20] Devaraj A, Perea DE, Liu J, Gordon LM, Prosa TJ, Parikh P, Diercks DR, Meher S, Kolli RP, Meng YS, Thevuthasan S (2017). Three-dimensional nanoscale characterisation of materials by atom probe tomography. Int. Mater. Rev..

[21] Tsong TT, Müller EW (1970). Field adsorption of inert-gas atoms on field ion emitter surfaces. Phys. Rev. Lett..

[22] Tsong TT, Müller EW (1971). Field adsorption of inert-gas atoms. J. Chem. Phys..

[23] Thompson K, Lawrence D, Larson DJ, Olson JD, Kelly TF, Gorman B (2007). In situ site-specific specimen preparation for atom probe tomography. Ultramicroscopy.

[24] Miller MK, Russell KF, Thompson K, Alvis R, Larson DJ (2007). Review of atom probe fib-based specimen preparation methods. Microsc. Microanal..

[25] Kolli RP, Meisenkothen F (2014). A focused ion beam specimen preparation method to minimize gallium ion concentration in copper atom-probe tomography specimen tips. Microsc. Microanal..

[26] Thompson K, Gorman B, Larson D, van Leer B, Hong L (2006). Minimization of Ga induced FIB damage using low energy clean-up. Microsc. Microanal..

[27] Saxey DW (2011). Correlated ion analysis and the interpretation of atom probe mass spectra. Ultramicroscopy.

[28] Meisenkothen F, Steel EB, Prosa TJ, Henry KT, Prakash Kolli R (2015). Effects of detector dead-time on quantitative analyses involving boron and multi-hit detection events in atom probe tomography. Ultramicroscopy.

[29] Kolli RP, Meisenkothen F (2014). Optimization of experimental parameters and specimen geometry for pulsed laser atom-probe tomography of copper. Microsc. Microanal..

[30] Kolli RP, Meisenkothen F (2014). The influence of experimental parameters and specimen geometry on the mass spectra of copper during pulsed-laser atom-probe tomography. Microsc. Microanal..

[31] Vurpillot F, Houard J, Vella A, Deconihout B (2009). Thermal response of a field emitter subjected to ultra-fast laser illumination. J. Phys. D Appl. Phys..

[32] Kellogg GL, Tsong TT (1980). Pulsed-laser atom-probe field-ion microscopy. J. Appl. Phys..

[33] Kellogg GL (1981). Determining the field emitter temperature during laser irradiation in the pulsed laser atom probe. J. Appl. Phys..

[34] Cerezo A, Smith GDW, Clifton PH (2006). Measurement of temperature rises in the femtosecond laser pulsed three-dimensional atom probe. Appl. Phys. Lett..

[35] Cerezo A, Clifton PH, Gomberg A, Smith GDW (2007). Aspects of the performance of a femtosecond laser-pulsed 3-dimensional atom probe. Ultramicroscopy.

[36] Vurpillot F, Gault B, Vella A, Bouet M, Deconihout B (2006). Estimation of the cooling times for a metallic tip under laser illumination. Appl. Phys. Lett..

[37] Forbes RG (1989). On charged-surface models and the origin of field adsorption. Surf. Sci..

[38] Wang RLC, Kreuzer HJ, Forbes RG (1996). Field adsorption of helium and neon on metals: an integrated theory. Surf. Sci..

[39] Forbes RG (1991). Further comments on field adsorption. Surf. Sci..

[40] Knickelbein MB (2004). Electric dipole polarizabilities of copper clusters. J. Chem. Phys..

[41] Slater JC (1964). Atomic radii in crystals. J. Chem. Phys..

[42] Madhavan PV, Whitten JL (1981). Hydrogen adsorption on copper: embedding theory based on orbital localization. Surf. Sci..

[43] Forni A, Wiesenekker G, Baerends EJ, Tantardini GF (1994). The chemisorption of hydrogen on Cu(111): a dynamical study. Int. J. Quantum Chem..

[44] Ye X, Kreuzer HJ, Salahub DR (1993). Theory of field adsorption of hydrogen. Appl. Surf. Sci..

[45] Reckzügel MC, Drachsel W, Block JH (1994). Field desorption of H_3_ and field dissociation of H^3+^. Appl. Surf. Sci..

[46] Tsong TT, Kinkus TJ, Ai CF (1983). Field induced and surface catalyzed formation of novel ions : a pulsed-laser time-of-flight atom-probe study. J. Chem. Phys..

[47] Ai CF, Tsong TT (1984). A study of the temperature dependence of a surface catalyzed and field enhanced formation of H_3_ and NH_3_ on metal surfaces. J. Chem. Phys..

[48] Ai CF, Tsong TT (1984). Field promoted and surface catalyzed formation of H_3_ and NH_3_ on transition metal surfaces: a pulsed-laser imaging atom-probe study. Surf. Sci..

[49] Ernst N, Block JH (1983). Temperature programmed field desorption of protonated hydrogen from rhodium and tungsten. Surf. Sci..

[50] Choi P-P, Cojocaru-Mirédin O, Abou-Ras D, Caballero R, Raabe D, Smentkowski VS, Park CG, Gu GH, Mazumder B, Wong MH, Hu Y-L, Melo TP, Speck JS (2012). Atom probe tomography of compound semiconductors for photovoltaic and light-emitting device applications. Microsc. Today.

[51] Diercks DR, Gorman BP (2015). Nanoscale measurement of laser-induced temperature rise and field evaporation effects in CdTe and GaN. J. Phys. Chem. C.

